# Investigating the Configurations in Cross-Shareholding: A Joint Copula-Entropy Approach

**DOI:** 10.3390/e20020134

**Published:** 2018-02-20

**Authors:** Roy Cerqueti, Giulia Rotundo, Marcel Ausloos

**Affiliations:** 1 Department of Economics and Law, University of Macerata, via Crescimbeni, 20, Macerata 62100, Italy; 2 Department of Statistical Sciences, Sapienza University of Rome, p.le A. Moro 5, Roma 00185, Italy; 3 School of Business, University of Leicester, University Road, Leicester LE1 7RH, UK

**Keywords:** entropy, cross-shareholdings, concentration, copulas

## Abstract

The complex nature of the interlacement of economic actors is quite evident at the level of the Stock market, where any company may actually interact with the other companies buying and selling their shares. In this respect, the companies populating a Stock market, along with their connections, can be effectively modeled through a directed network, where the nodes represent the companies, and the links indicate the ownership. This paper deals with this theme and discusses the concentration of a market. A cross-shareholding matrix is considered, along with two key factors: the node out-degree distribution which represents the diversification of investments in terms of the number of involved companies, and the node in-degree distribution which reports the integration of a company due to the sales of its own shares to other companies. While diversification is widely explored in the literature, integration is most present in literature on contagions. This paper captures such quantities of interest in the two frameworks and studies the stochastic dependence of diversification and integration through a copula approach. We adopt entropies as measures for assessing the concentration in the market. The main question is to assess the dependence structure leading to a better description of the data or to market polarization (minimal entropy) or market fairness (maximal entropy). In so doing, we derive information on the way in which the in- and out-degrees should be connected in order to shape the market. The question is of interest to regulators bodies, as witnessed by specific alert threshold published on the US mergers guidelines for limiting the possibility of acquisitions and the prevalence of a single company on the market. Indeed, all countries and the EU have also rules or guidelines in order to limit concentrations, in a country or across borders, respectively. The calibration of copulas and model parameters on the basis of real data serves as an illustrative application of the theoretical proposal.

## 1. Introduction

The recent crises have evidenced the fragility of the financial system due to the growing interdependencies among many different organizations.

In the context of network modeling applied to management organizations of industrial structures, usually nodes represent companies, while the links show the ownership, gathered in the cross-shareholding matrix. However, many studies in literature mostly focused on the shape of the distribution of the node out-degree $k_{out}$, because such results are linked to specific results on the resilience of the network [1,2,3,4,5]. $k_{out}$ represents the number of the companies whose stocks are included in the portfolio of the considered company, i.e., it is the amount of different counterparts. Therefore, $k_{out}$ can be used for representing the diversification, according to its conceptualization in the reference literature (see e.g., [6]). The higher the diversification, the less sensitive the node is to its inner fluctuations.

Surprisingly, not many studies were done on the node in-degree $k_{in}$ distributions, where $k_{in}$ is the amount of (other) companies who bought some ownership of a specific company. The in-degree well represents the way in which each organization becomes more dependent on its counterparts, so it can be used to represent the integration of the company in the system (also for the concept of integration, refer to [6]).

Notice that the construction of $k_{out}$ and that of $k_{in}$ do not involve the entity of the connections among companies, but only the number of existing connections. Thus, such quantities serve for modeling the presence of interactions; this provides information on how a company is integrated in the system and how diversified is its portfolio.

An initial increase of integration may allow financial fluctuations of the value of a company to propagate and very high integration allows eventual cascades to spread on so many units that its effects are minimal [7]. Literature contributions inquired furthermore on the trade-off among integration and differentiation so to detect the most dangerous combination for the propagation of a global crisis [7]. In this respect, it is also worth mentioning other ways for interconnections among companies, like the interlock of directorates [8,9,10], technological transfer [11] personal relationships [12,13,14], organizational capabilities [12,15] or other contractual relationship (for a survey, see [16]). In this respect, a special mention should be done for systemic risk models [17,18].

However, it is important to stress once again that $k_{out}$ has been studied more than $k_{in}$ in the empirical literature (see the review below).

Studies on different real world networks have shown different reactions to patterns of attack among highly versus low concentrated networks. In short, highly concentrated networks are resilient to random shocks, but most sensitive to attacks to the core and to hubs. On the opposite, low concentrated networks are sensitive to random attacks [19,20,21] or exhibit peculiar structural characteristics when combined with the features of the nodes [22].

In this paper, we elaborate on the market concentration, represented through the entropies of the distributions of diversification and integration. In a connected network, under the hypothesis of independence among $k_{in}$ and $k_{out}$, the entropy is minimal when the $k_{in}$ is concentrated on one value only; the same happens for $k_{out}$. For instance, this happens on lattices or regular grids. Apart from being quite unlikely as cross-shareholding configuration, empirical evidences in literature assess the power law for the probability of $k_{out}$. Moreover, there is evidence also on a power law or exponential behavior for the probability distribution of $k_{in}$, as it is going to be detailed in the next section. Such distributions are discrete and on a limited range of integer numbers. In principle, these shapes of the marginal distributions of the in- and out-degrees should prevent the achievement of the minimum of the entropy, of course unless the joint structure is not the independent, but an ad-hoc one. It could also happen that—although keeping the power law/exponential form—the measures are so concentrated on their center of mass that the entropy is quite close to its minimum. In this case, most of the network units should have just one incoming and one outgoing link; that is, again, a very unlikely configuration for a cross-shareholding network. On the opposite, the maximum level of concentration increases when there is a flat uniform distribution. In this case – in order to make an example – again under the hypothesis of independence – the units with the minimum $k_{in}$ should have the maximal $k_{out}$; and vice versa (see the Appendix A for further insights). This situation is much closer to the kind of networks modeling the presence of mixed categories of companies. In fact, usually financial companies land money in exchange of shares; but sell their shares to a minimal number of other companies, maximum one or two [23]. On the opposite, manufactures sell their shares, but rarely make financial investments buying shares of other companies—unless strategically relevant to their specific business [23,24].

In front of such different landscapes, some main research question addressed in the present paper is exactly on these topics: is the hypothesis of independence holding on a case study? Is the network topology of the case study limited to the distribution of $k_{in}$ and $k_{out}$ sufficient, in itself, to prevent a rise of concentration? Would there be maxima/minima of the entropy if - keeping the marginals - the joint structure would be different? To which extent may the parameters describing the marginals change before eventually reaching maximum or minimum of concentration?

In order to achieve the tasks, we adopt a copula approach for assessing the concentration of the market through the stochastic dependence between in- and out-degree. In this respect, copulas are of great usefulness (see [25,26]). Indeed, the classical Sklar’s Theorem [27] explains that a copula function is able to represent the connection between the joint probability distribution of a random vector and the marginals of its components. Specifically, a multivariate copula computed over the marginals is equivalent to the joint distribution. Sklar’s Theorem can also be read under a different perspective: starting from a joint distribution of a random vector and the marginals of its components, one can implement a best fit procedure to identify the copula describing the connection among them.

Thus, as already stated above, concentration is here captured through the joint analysis of diversification and integration at an aggregate level. Specifically, it is given by the Shannon entropy of the joint distribution of in- and out-degree. This leads to gain insights on the market structure and on other relevant aspects, like the reaction of the system to external shocks. Indeed, a polarized market (minimum value of the entropy) can be associated to the presence of a company with a central role, while a large entropy suggests a fair distribution of the business network in terms of companies ownerships.

It is worth remarking that a proper consideration of the weights of the network would make entropy equivalent to the Herfindahl–Hirschman (HH) measure of concentration, that became quite popular in financial studies after its appearance in the official documents of the US mergers guidelines for fixing alert threshold [28].

The present study offers to the regulatory bodies the possibility to monitor the rise of concentration by looking only to the network topology and to the stochastic dependence between in- and out-degree.

For what concerns the dependence structure of diversification and integration, we proceed under two different perspectives. By one side, we consider the independence copula and the Frechet bounds [29], which are specific fundamental nonparametric copulas, and assume that they describe the dependence between the two degrees random variables. On the other hand, we calibrate the parameters of three families of copulas—Gumbel, Clayton and Frank, see [30,31,32], respectively—which belong to the classical family of Archimedean copulas [33].

In so doing, we focus on the informative content of the stochastic dependence between in- and out-degree random variables. In fact, the different copulas capture different stochastic dependence among the involved random variables. In particular, Frechet bounds have an intuitive interpretation in the bivariate case: they represent the maximum absolute values of joint correlations. The upper bound stands for the highest positive correlations, while the lower one is for negative correlations. The Gumbel copula captures tail dependence, with a special attention towards the dependence on the right tail. Differently, The Clayton copula [30] describes the dependence on the left tail of the distribution. Frank copula [31] does not exhibit tail dependence and allows both positive and negative dependence.

The methodology used for the calibration procedure is based on two different optimization problems, i.e., a maximum- and minimum-entropy for the joint distribution. In the former case, we are in the corner situation of an economic system with companies having the same values of diversification and integration; the latter case is associated to the maximum level of polarization, with only one company holding the total amount of connections, so that the maximum level of diversification and integration.

In the same light, entropy is also computed in the case of nonparametric copulas for the obtained multivariate joint distribution. The paradigmatic cases of independence—product copula—and maximum/minimum level of positive dependence—the Frechet bounds—serve as benchmarks.

The analysis has been also expanded for including a generic economic system. Indeed, many empirical papers evidenced that the distribution of the out-degree of many economic-financial systems is of a power law type [34]. Thus, the analysis has been replicated by substituting the out-degree index with a power law function. The parameter of the power law has been included in the set of parameters to be calibrated. The empirical evidences on both the existence of power law and of the exponential distribution for the in-degree will be examined as well.

The generalization of the results of this paper to other kind of networks, such as networks with missing links, is challenging and useful. We have in mind contributions on not fully observable networks that can be effectively adopted (see e.g., [35,36,37]); this topic might be some matter for future work.

The rest of the paper is organized as follows: the next sections describe the selection of the probability distribution of the marginals according to the existing literature and empirical data. Section 3 presents the employed dataset. Section 4 outlines the investigation procedure along with the considered copulas. Section 5 contains the obtained empirical results on the case study and on the generalizations and discusses them. Last section concludes. Some important ancillary results and materials are relegated in two devoted Appendices.

## 2. Distribution of the in- and out-Degrees: Empirical Evidences in Literature and a Case Study

This section serves to fix the hypotheses on the shapes of the marginal distribution that are meaningful for the problem under examination.

In literature—most in the Econophysics realm—there was much emphasis in the detection of the Pareto distribution in Economics [38,39]. Such a distribution is characterized by a power law decay in the tails:

(1) $$p\left(k\right)∼k^{-\gamma }$$

that corresponds to the cumulative distribution

(2) $$P\left(k\right)∼k^{1-\gamma }$$

Therefore, if *k* follows a power law with the exponent −γ, then the cumulative distribution function P(k) follows the power law with exponent −γ+1.

### 2.1. The out-Degree $k_{out}$

The presence of the power law in the distribution of the out-degree is widely assessed in existing literature.

For example, Aoyama et al. [6,40] add evidences to the power law of the out-degree analyzing the shareholding network of Japanese companies listed in the Japanese stock market by using only major shareholder data, and focusing on companies concerned with automobile manufacture. The results reported (see Figure 4.28 and Table 4.5 in [40]) show the analysis of the cumulative distribution of outgoing degrees in 1985, 1990, 1995, 2000, 2002, and 2003. The size of the dataset ranges from 2078 to 3770 companies, and all annual cumulative distributions can be well fitted by a power-law distribution with exponents in the range (1.67,1.86), that leads to γ∈(2.67,2.86).

Souma et al. [41] examine the Japanese shareholding network existing at the end of March 2002. The network is constructed from 2303 listed companies and 53 non listed financial institutions. The distribution of outgoing degrees is well explained by the power law function with an exponential tail. The best fit of the cumulative is a power law with exponent 1.7, that corresponds to γ=2.7.

In [42], the direction of links reversal to the one used in [8,23,43] is used for dealing with diversification and integration, so their results for $k_{in}$ actually have to be compared with $k_{out}$ of the other papers. The authors report also the power law exponents of some shareholding networks: the Italian stock market (Milano Italia Borsa; MIB), the New York Stock Exchange (NYSE), and the National Association of Security Dealers Automated Quotations (NASDAQ). They find that all of them follow a power law distribution: $\gamma_{MIB}=2.97$ in 2002, $\gamma_{NYSE}=2.37$ in 2000, $\gamma_{NASDAQ}=2.22$ in 2000.

The scale free structure has been estimated also on the shareholding of 223 companies quoted in MIB (Milan Stock Exchange) in the time span 1/1/2004, 12/31/2004 [43]. Companies are the network nodes; arcs are drawn from the shareholders to the owned companies. The power law function with exponent 1.39, that leads to γ=2.39 nicely fits the distribution.

In [23] the shareholding network of MIB companies are still built as in [43], but on data sampled in 2008. A best fit estimate of 2.15 and a Maximum Likelihood Estimate of γ=2.7, are in line with the above mentioned results.

In [44] the cross-shareholding of 300 index companies from 2007 to 2013 are studied. The companies are listed in the Shanghai and Shenzhen stock market. Data are provided by the Securities Times (STCN) and the Wind Database. The sample of firms covers about sixty percent of the market value of the Shanghai and Shenzhen stock market. They find the following values of γ: γ=2.311 (2007), γ=2.465 (2008), γ=2.558 (2009), γ=2.625 (2010), γ=2.721 (2011), γ=2.722 (2012), γ=2.724 (2013).

In [45] the worldwide network of listed energy companies sampled in 2013 is built. The data source is the ORISE publicly listed companies worldwide (https://osiris.bvdinfo.com), on December 31, 2013. There are 2334 listed energy companies and 8302 shareholders in the database (after removing duplicate items). In this so large database, the power law exponent estimated for the cumulative distribution of the out-degree is γ=2.428.

In [46] the cross-shareholding networks of the companies listed in Chinese stock market between 2002 and 2009 are studied. They analyze the mutual investment at company-level, province-level and region-level. However, they go beyond the mere topology of the network, because they consider the weight of cross-ownerships into the out-degree. Although they measure a quantity different from the $k_{out}$ that we use in this paper, it is worth remarking that they measure the power law in the range (1.813−2.229). In details: 2.229 (2002), 2.152 (2003), 2.057 (2004), 1.958 (2005), 1.899 (2006), 1.788 (2007), 1.793 (2008), 1.813 (2009).

The topological properties and evolution of the cross-shareholding networks of listed companies Shanghai stock exchange and the Shenzhen stock exchange in China from 2007 to 2011 are analyzed in [47]. They find that both the in-degree and the out-degree follow a power law distribution in the range (2.01,2.43). In details: 2.43 (2007), 2.39 (2008), 2.33 (2009), 2.32 (2010), 2.33 (2011).

Vitali et al. [48] worked on the Orbis 2007 marketing database, that comprises about 37 million economic actors, both physical persons and firms located in 194 countries, and roughly 13 million directed and weighted ownership links (equity relations). On such data, the power-law exponent of the probability density function of the out-degree is γ=2.15.

We may conclude that above empirical analyses allow to conclude that the power law behavior of $k_{out}$ is quite widespread, and allows us to assume a power law as hypothesis for $k_{out}$.

### 2.2. The in-Degree $k_{in}$

The amount of empirical analyses of $k_{in}$ is much lower than the ones on $k_{out}$. Some authors explicitly declare that they are not interested in examining $k_{in}$, because the range of this variable is more limited than $k_{out}$. A very few studies are available. In [43] the in-degree distribution shows a power law, with exponent 0.62. On [23] data, the exponential distribution was detected as the best fitting one, although the power law is quite close. Therefore, we are going to examine both the power law and the exponential as probabilities suitable for describing $k_{in}$.

## 3. Data

The data is the set of holdings among listed firms in the Milan Stock Market. It is the same as in [23]. The data set has been sampled on May 10th, 2008, from which we build the network of shareholders and subsidiaries of companies traded on the MTA segment *www.borsaitaliana.it/azioni/mercati/mta/.../mta-mercato-telematico-azionario.en.htm* of the Italian Stock Market. The information available on several databases were cross-checked: the Bureau Van Dijk databases and CONSOB for the active and passive ownership sample; Bankscope for banking and financial companies; ISIS for insurance companies; AIDA for all the remaining sectors; Datastream Thomson Financial Database. The few companies that had incomplete data on either active or passive holdings were excluded from the present analysis. Analogously, we have excluded also the non-listed companies, since reliable data on them are not available. Even if very limited holdings (below 2%) have been considered, the mediate possessions held via mutual funds were excluded as well, because they do not represent a direct interest of a company into another.

The total size of the sample amounts to 247 companies, that represent the nodes of the network, that is the 94% of the total number of listed companies and 95.22% in terms of capitalization. This dataset is slightly different from the one examined in Garlaschelli et al. (2005) because some companies traded in the market changed; moreover, there is a different level of accuracy in the details of ownership data, and their $k_{in}$ corresponds to our $k_{out}$. Our notation for $k_{out}$ is following [49].

Most companies do not actually buy shares of other companies, they can be considered small companies. The giant component is made by 101 nodes, which are connected to each other [23]. In the present analysis, we consider only the values of the in-degree and of the out-degree that are different from 0, so that we exclude isolated nodes. The latter constitute the set of companies that do not buy shares of (and which shares are not owned by) other companies traded in the same market.

## 4. Investigation Procedure

This section is devoted to the introduction of the analytical instruments used and to the description of the implemented analysis.

### 4.1. The Adopted Copulas

We firstly present the definition of bivariate copula, which is crucial for the study.

**Definition** **1.** *A bivariate copula is a function $C:{\left[0,1\right]}^{2}\to \left[0,1\right]$ such that*

- C(u,v)=0 if u×v=0;
- C(u,1)=u and C(1,v)=v, for each u,v∈[0,1];
- *Given the 2-dimensional rectangle $\left[a_{1},b_{1}\right]\times \left[a_{2},b_{2}\right]\subseteq {\left[0,1\right]}^{2}$, then*
  $$\sum_{i_{1}=1}^{2}\sum_{i_{2}=1}^{2}{\left(-1\right)}^{i_{1}+i_{2}}C\left(u_{i_{1}},v_{i_{2}}\right)\geq 0,$$
  *where $u_{j}=a_{j}$ and $v_{j}=b_{j}$.*

The concept of bivariate copula plays a key role in describing the stochastic dependence between two random quantities. Such a statement is formalized in the Sklar (1959)’s Theorem, reported below:

**Theorem** **1.** *Let P be the joint distribution function of a bivariate random variable (X,Y). Define the margins as $P_{X}$ and $P_{Y}$. Then there exists a bivariate copula C such that, for each $\left(x,y\right)\in R^{2}$,*

(3) $$P\left(x,y\right)=C(P_{X}\left(x\right),P_{Y}\left(y\right)).$$

*If the margins $P_{X},P_{Y}$ are continuous, then the copula C is unique. Conversely, if C is a bivariate copula and $P_{X},P_{Y}$ are distribution functions, then the function P defined in (3) is a bidimensional distribution function with margins $P_{X},P_{Y}$.*

Theorem 1 explains that the relationship between the joint and the marginal distributions of a couple of random variables can be formalized by employing copulas.

Different copulas describe different types of stochastic dependence. The analysis here implemented refers to six copulas—or classes of copulas—which are widely used in the applications.

Specifically:

- Product copula
  (4) $$C_{I}\left(u,v\right)=uv.$$
  This is the case in which the random variables *X* and *Y* are independent.
- Lower Frechet bound
  (5) $$C_{LF}\left(u,v\right)=\max\left\{u+v-1,0\right\}$$
  This copula represents the case of perfect negative correlation between *X* and *Y*.
- Upper Frechet bound
  (6) $$C_{UF}\left(u,v\right)=\min\left\{u,v\right\}$$
  This copula, in an opposite way with respect to the previous one, captures perfect positive correlation between *X* and *Y*.
- Gumbel Archimedean copula
  (7) $$C_{G}\left(u,v\right)=\exp\left[-{\left({\left(-\ln\left(u\right)\right)}^{\theta }+{\left(-\ln\left(v\right)\right)}^{\theta }\right)}^{1/\theta }\right],\;\theta \in \left[1,+\infty \right)$$
  In this case, one has an asymmetric tail dependence, with more mass on the right tail. Such a dependence is influenced by the value of the parameter θ.
- Clayton Archimedean copula
  (8) $$C_{C}\left(u,v\right)={\left[\max\{u^{-\theta }+v^{-\theta }-1,0\}\right]}^{-1/\theta },\;\theta \in \left[-1,0\right)\cup \left(0,+\infty \right)$$
  Analogously to the previous case, here one has an asymmetric tail dependence. However, Clayton copula is associated to a predominance of the left tail.
- Frank Archimedean copula
  (9) $$C_{F}\left(u,v\right)=-\frac{1}{\theta }\ln\left[1+\frac{\left(\exp(-\theta u)-1)(\exp(-\theta v)-1\right)}{\exp(-\theta )-1}\right],\;\theta \neq 0$$
  This copula is not associated to tail dependence, and is able to capture either positive or negative dependence on the basis of the value of θ.

Product copula and the Frechet bounds are associated to nonparametric functions, since they do not depend on any parameter. Differently, the presence of a scalar θ in the definition of Gumbel, Clayton and Frank copula says that such copulas are of parametric type.

### 4.2. Outline of the Analysis and Numerical Results

The availability of the case study allows to have a full description of the marginals and of the joint distribution of the in- and out-degrees. However, the general case is also included for the sake of universality of the analysis.

The investigation procedure is split in three cases. In all the steps, the above-mentioned copulas are taken as reference instruments, in order to describe stochastic dependence between the in- and the out-degree and achieve different objectives.

In the case 1, a description the empirical data coming out from the available sample is provided. Starting from the empirical (marginal) distributions of in-degree and out-degree, we derive the joint distribution of such quantities by applying Sklar (1959)’s Theorem through the copulas introduced above. The Euclidean distance between the non-parametric copula-based distributions are computed, and also the calibration of the parameters of the Archimedean copulas are obtained by a Euclidean distance minimization.

Case 2 still focuses on the case study. Substantially, this step can be viewed as a replication of the previous one with the remarkable difference that the Euclidean distance has been replaced by the Shannon entropy. The meaning of this second step of the analysis can be easily synthesized. Indeed, we here look at the conditions on the stochastic dependence between in- and out-degrees leading to market polarization (minimal entropy) or market fairness (maximal entropy). In so doing, we derive information on the way in which the degrees should be connected in order to shape the market. Two separate cases are treated: first, computation of the entropy for the cases of non-parametric copulas; second, the calibration of the parameters of the considered Archimedean copulas under a maximum- and minimum-entropy approach.

In the case 3, we provide a generalization and, in accord to the existing literature, we consider marginal densities depending on parameters. In details, we consider power-law and exponential for the out-degree, while we take the in-degree without parametrization, according to its empirical distribution. Also in this case, two cases are treated: first, the non-parametric copulas are imposed and the parameters of the power laws and exponential are calibrated under a maximum- and minimum-entropy approach; second, the parametric copulas of Gumbel, Frank and Clayton types are considered and their parameters, along with that of the out-degree distribution, are calibrated in a max/min entropy approach.

The probability of configuration $P(k_{in}=i,k_{out}=j)$ is calculated through the copula as $P\left(k_{in}=i,k_{out}=j\right)=C\left(u\left(i\right),v\left(j\right)\right)-C\left(u\left(i-1\right),v\left(j\right)\right)-C\left(u\left(i\right),v\left(j-1\right)\right)+C\left(u\left(i-1\right),v\left(j-1\right)\right)$.

Moreover, the calibration methods might naturally be based on other concepts of distance (see e.g., [50,51]). In this respect, it is also worth mentioning the results and methodologies proposed in Schellcase (2012), where the author provides an estimation of copula density through penalized splines of different types [52]. However, as already pointed out above, Euclidean distance and entropy have different meanings and are particularly suitable for capturing the focuses of our investigation purposes.

## 5. Results and Discussion

The obtained findings of the analysis are here described and discussed.

### 5.1. Case 1: Distance from the Empirical Joint Distribution

**Figure 1:**
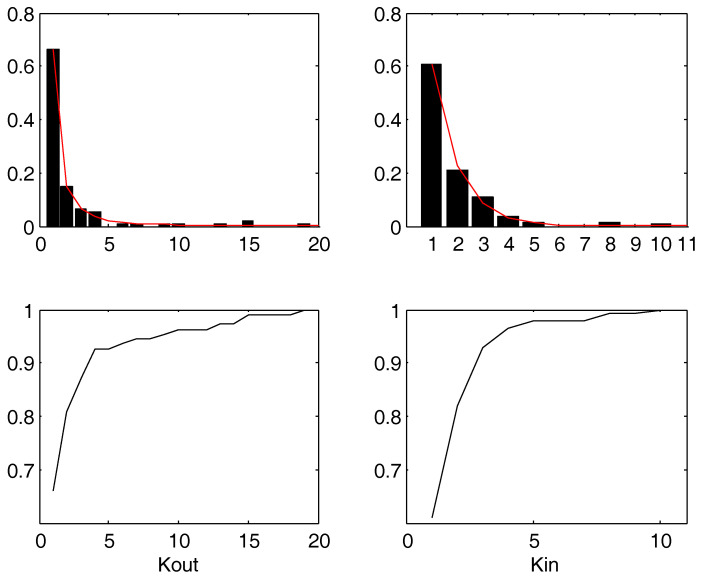
Upper figures: histograms (empirical densities, left: $p(k_{out}=x)$, right: $p(k_{in}=x)$). Lower figures: distributions (left: $P(k_{out}<x)$, right: $P(k_{in}<x)$). The left part corresponds to Figure 4 of [23].

**Figure 2:**
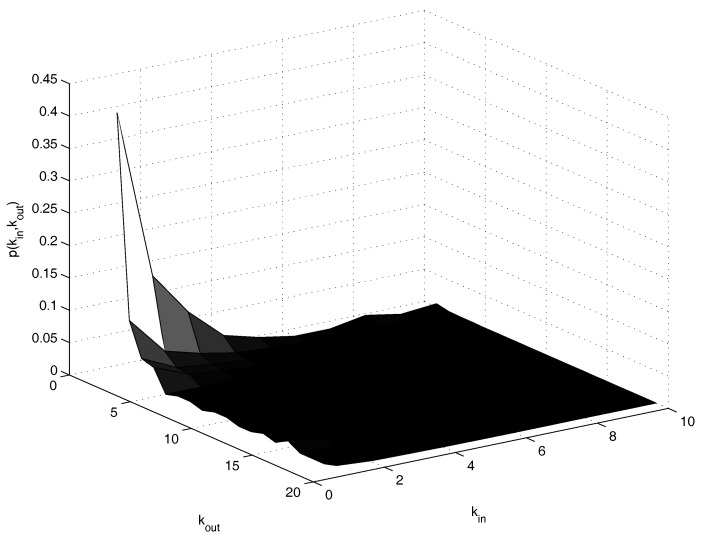
Case study. Joint empirical distribution.

Figure 1 shows the empirical marginal distribution of $k_{in}$ and $k_{out}$ for the empirical case we deal with, while Figure 2 shows the joint probability. The range for $k_{in}$ is [1,⋯,10], and the range for $k_{out}$ is [1,⋯,19]. The limits of 10 for *i* and 19 for *j* are due to the specific sample. The value 0 is not considered in the present analysis. In fact, the detection of the Pareto distribution would mainly concern the tails. Thus, we notice that there are too many 0’s for appreciating such a distribution in the full histogram.

The power law best fit over the density gives $p\left(k_{out}\right)∼k_{out}^{-\gamma }$ with γ=2.159(1.984,2.339), RMSE = 0.0094. The Jarque-Bera test validates the hypothesis of Gaussianity of residuals. The power law best fit on the empirical probability distribution leads to $P\left(k_{out}\right)∼k^{1-\gamma }$ where γ=1.7925(1.6596,1.9254), RMSE = 0.0088. The MLE γ gives γ=2.72766(2.72763,2.72768). For the case of in-degree, the Jarque-Bera test rejects the hypothesis of Gaussianity of residuals. Therefore, there is still residual information in the residuals whence the hypothesis of power law decay cannot be fully validated. However, the empirical distribution is quite close to the power law. For the in-degree $k_{in}$ the best fit is the exponential General model Exp1: f(x)=a·exp(b·x) Coefficients (with 95% confidence bounds): *a* = 1.6 (1.424, 1.777) *b* =−0.9727 (−1.061, −0.8845) Goodness of fit: SSE: 0.001137 R-square: 0.9966 Adjusted R-square: 0.9963 RMSE: 0.01124.

The parametric copula—Gumbel, Frank and Clayton—that best fits to the empirical data is now detected. For the non parametric copulas we calculate the distance $d(C_{I},P)$ of the joint distribution calculated by using the copula C(u,v) from the empirical joint distribution *P*. Such a distance will be used as a benchmark value.

The results are:

- Product copula (independence): $d(C_{I},P)=4.06e-014$
- Lower Frechet bound $d(C_{LF},P)=0.9354$
- Upper Frechet bound $d(C_{UF},P)=3.9484$

Therefore, the joint empirical distribution is closer to the hypothesis of independence (product copula) than to the others.

**Figure 3:**
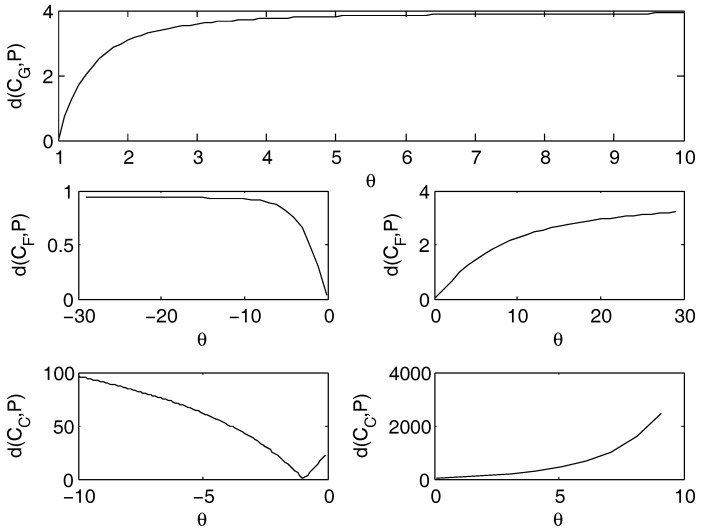
Distance d(C,P) from the empirical distribution, when the joint distribution is calculated through the Gumbel (upper figure, $d(C_{G},P)$), Frank (middle figures, $d(C_{F},P)$) or Clayton distribution (lower figures, $d(C_{C},P)$).

On the copulas that depend on a parameter a best fit procedure has been implemented. Figure 3 plots the dependence of the distance on θ considering the three cases for the joint distribution: the Gumbel, Frank and Clayton copulas:

- Gumbel Archimedean copula. The best fit holds for θ=1, with practically 0 as value for the distance. This is coherent with the case of the product copula, because, in fact, when θ=1, then the Gumbel copula reduces to the product copula. Small differences on the distance are due to the numerical rounding of the algorithm. This outcome confirms what obtained for the independence case.
- Frank Archimedean copula. The distance from the empirical data is decreasing as θ approaches 0, but 0 does not belong to the definition set. Therefore, the calibrated parameter tends to zero. We do not have an optimal value of θ. From this, we infer that this copula is not suitable for the fit.
- Clayton Archimedean copula. For the negative values of θ, there is a minimum for θ=−1, that belongs to the definition set and corresponds to the case of the lower Frechet bound. The value of the distance for θ=−1 is 0.93.

Thus, the empirical in- and out-degrees exhibit a structure of stochastic independence, with a very small value of the distance between the empirical distribution and the one obtained in the product copula case. This is also confirmed in the Gumbel copula case. However, when forced to describe a type of dependence described through a Clayton copula, data are less distant from an absolute negative correlation (lower Frechet bound). This outcome is in agreement with the fact that the distance of the data from the lower Frechet bound is lower than the one from the upper Frechet bound.

Under an economic point of view, independence means that there is not a regular behavior of companies in the respect of integration and diversification. More precisely, it is not possible to infer diversification properties of the market by looking at the integration, and vice versa.

### 5.2. Case 2: Entropy

In this section, we start working on the entropy. We refer to the Shannon entropy [53]

(10) $$H\left(C\left(u,v,\theta \right)\right)=-\sum_{u,v}C\left(u,v,\theta \right)\ln C\left(u,v,\theta \right)$$

The entropy calculated on the empirical joint distribution is 1.52. On the joint distribution calculated through the copulas not depending on parameters, the values of the entropy are:

- Product: H=1.52, the same value as for the empirical joint distribution. In fact, this copula well describes the joint distribution.
- Lower Frechet: H=0.96.
- Upper Frechet: H=1.45.

**Figure 4:**
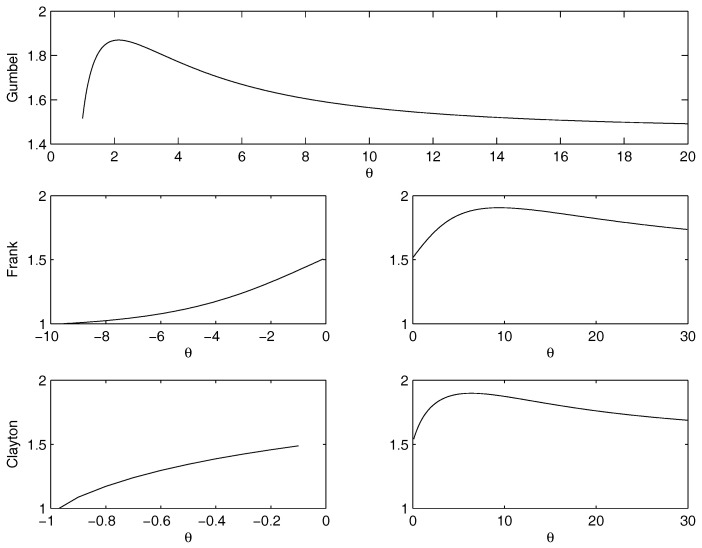
Plot of the dependence of the entropy function on the parameter theta for the Gumbel, Frank and Clayton, calculated on the marginals of the case study. Clearly, no minimum internal to the definition sets. There is a maximum for the Gumbel copula in θ=2.13. There is a maximum for the Frank copula in θ=9.41. There is a maximum for the Clayton copula in θ=6.39.

For the parametric copulas, we perform a comprehensive analysis on the minimum/maximum as a function of θ. Figure 4 shows the dependence of the entropy on θ in the cases of joint distribution calculated through copulas. We get the following results:

- Gumbel Archimedean copula. The numerical minimization procedure gives the best fit for θ=1, with a value of the entropy equal to 1.5154 . This is in line with the best fit of the product copula. From Figure 4 it is possible to note that there is an asymptotic behavior for θ going to infinity. The maximum is attained for θ=2.1312 with a value of the entropy equal to 1.8693.
- Frank Archimedean copula. There is no minimum because 0 does not belong to the definition set of the functions. The maximum is attained for θ=9.4205 with a value of the entropy equal to 1.9060.
- Clayton Archimedean copula. There is no minimum internal to the definition set. From Figure 4 it is clearly visible that the function is decreasing for θ<0, so θ=−1, that is the lower bound of the parameter variation interval, is a point of minimum. Regarding the maximum, the numerical maximization of the entropy gives the point of maximum in θ=6.3899, with a value of the entropy equal to 1.8982.

Results can be commented as follows. Independence is confirmed to describe the stochastic dependence between the degrees. More than this, we can also say that data are associated to a high value of the entropy. This outcome says that the market described by the considered companies has a "broadly fair" distribution in terms of integration and diversification. Such a "fairness" is more evident in the cases of Frank and Clayton copulas, whose calibrated parameters suggest that left tail dependence (Clayton) and positive correlation (Frank) are more likely associated to a uniform distribution of the in- and out-degrees. We point out that the left tail dependence is related to the presence of a strong correlation when the levels of diversification and integration are low.

The detection of a maximum shows that there are possible configurations for the joint distribution that lead to a network where the in-degree (distribution) is decoupled from the out-degree (distribution). Situations like this may happen when companies are artificially created, so that a wide set of combinations is possible: nodes with low (high) in-degree and high (low) out-degree or nodes with similar values of in-degree and out-degree. For instance, in the MIB30 ([23], Figure 1) the company IFI PRIV was created for controlling IFIL, that has the main role to provide financial services to the main companies of the Agnelli family: FIAT and JUVENTUS, so IFIL has only one outgoing link, and no incoming links - the ultimate owners being the persons member of the family. In [23], while Figure 2 in the quoted paper shows a list of companies for which the only link is due to the need of using a financial institution - that, in turn, gets ownership of the financed company. A circumstance that leads to quite different values for $k_{in}$ and $k_{out}$ for a single node is given by the role of banks and insurance companies: since they provide money to other companies, they get in exchange the ownership, whence having many outgoing links. On the other side, they use insurance companies transferring them their own part of their risk. In [23], Figure 1, on the left, the cases of MPS bank and UNIPOL insurance company clearly evidence this kind of situation.

### 5.3. Case 3: Marginals Depending on Parameters

The previous section has shown the case study. In literature, most often the $k_{out}$ follows a power law, with exponents in a range (2,3). The few studies on $k_{in}$ have shown most either a power law or an exponential. In this section, we aim at extending the previous results to a more general case in which the exponent of the power law may change. This corresponds to study the effect of a change of exponents on the results of the maximization and minimization of the entropy. It is worth recalling that the exponent of the power law has an implication on the presence of fair values. The higher the exponent, the faster is the decrease, meaning that there are many low values of the degrees and a very few with high ones. For instance, in [43] the MIB30 network of cross-shareholding was showing a power law. In fact, the companies considered in the quoted paper were more keen to diversify their investment. The crisis in 2008 canceled this kind of investment, as shown by the increase of the value of the power law exponent on the MIB30 in 2008 [23].

Although the power law remains the best fitting, the shape of the distribution is slowly moving to a sharply decreasing function, becoming closer to an exponential distribution. The same behavior of a distribution has been shown in [54] in the context of wealth.

For each of the above listed copulas, we here look for the minimal and maximal entropy using the following marginal distributions:

1. step 1: power law for $k_{out}$, and raw data for $k_{in}$.
2. step 2: raw data for $k_{out}$, and power law for $k_{in}$.
3. step 3: raw data for $k_{out}$, and exponential law for $k_{in}$.
4. step 4: power law for $k_{out}$, and power law for $k_{in}$.
5. step 5: power law for $k_{out}$, and exponential law for $k_{in}$.

The last two cases correspond to the most general case, independent from the case study. For each of them, all the copulas listed in the methodological section are tested.

To be concise and informative, we present here only step 1. The interested reader can find the other cases in Appendix B.

#### Step 1: Power Law for $k_{out}$, and Raw Data for $k_{in}$

In this case, we consider the cumulative distribution $P\left(k_{out}<x\right)=ax^{-k+1}$. We are not considering the more general functional form $ax^{-k+1}+b$ because the density in this kind of problems is vanishing as *k* increases, so *b* would be 0. The parameter *a* is automatically fixed by the normalization condition $P(k_{out}<\infty )=1$.

We already pointed out that the parameters regulate the mass distribution over the range. Low values of *k* lead to a more flat distribution; high values of *k* increase the skewness to the left, and so the cumulative distribution function is quickly growing at the beginning of the range; the inflectional point is moving to the left. The increase of the skewness leads to an alignment to the distribution of $k_{in}$, so increasing the peakness and the concentration of the distribution, hence the minimization of the entropy. Here below, we report results for both parametric and non parametric copulas. The figures referring to non-parametric copulas report *k* on the *x*-axis for the non parametric copulas. The parametric copulas depend on *k* and θ, but the 3D visualization is less clear than the 2D one. Therefore, the visualization for the parametric copulas is more clear drawing the entropy as function of θ (on the *x*-axis) for different meaningful values of *k* (corresponding to different curves).

**Figure 5:**
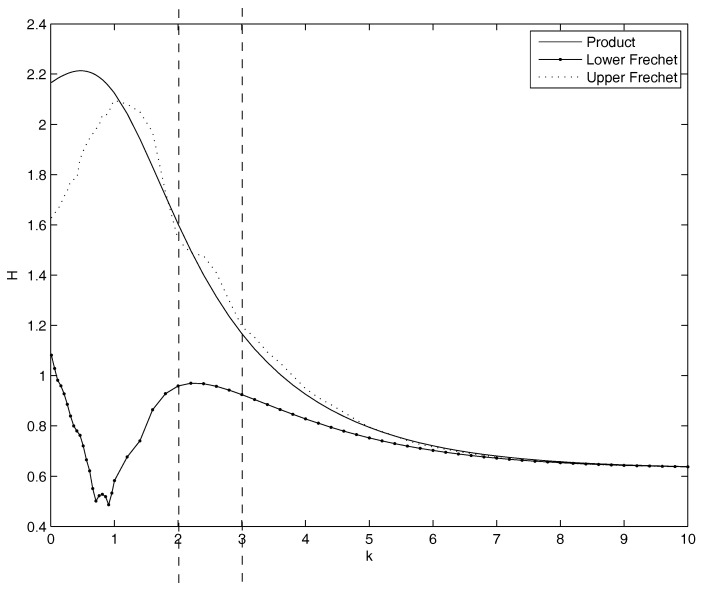
The figure shows the dependence of the entropy on *k* for each of the three non parametric copulas.

**Figure 6:**
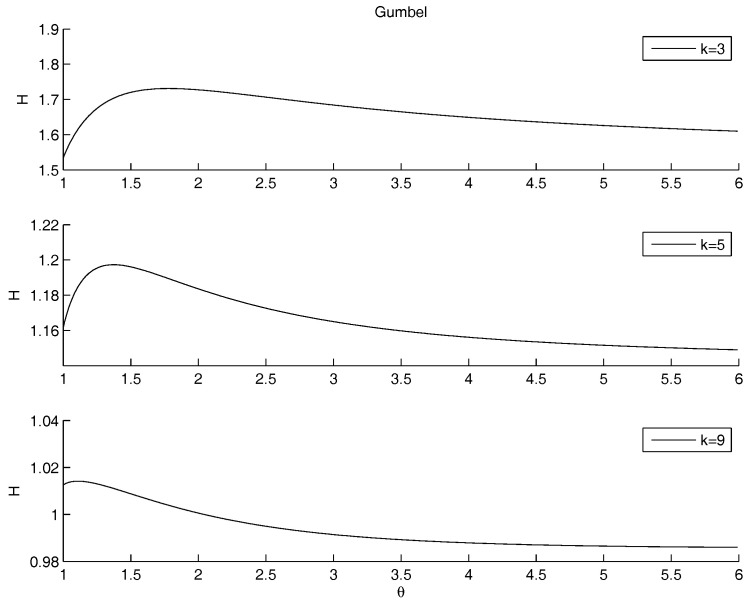
The figure shows three cases for the entropy (y-axis) as a function of θ (x-axis). The marginals are: the power law for $k_{out}$ and from the case study for $k_{in}$. They are combined through a Gumbel copula. In all cases, the function is decreasing as θ→∞. The maximum is well evidenced, like in our case study. As *k* increases, the maximum moves to the left border.

**Figure 7:**
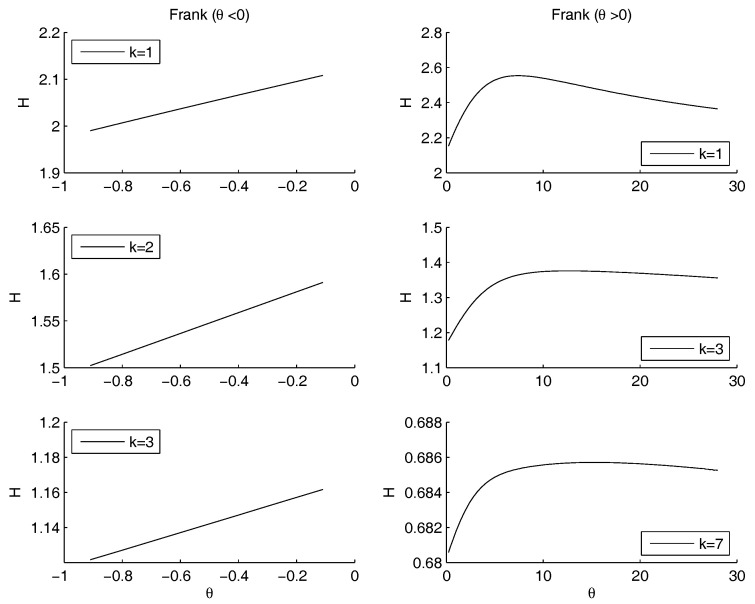
The figure shows three cases for the entropy (y-axis) as a function of θ (x-axis). The marginals are: the power law for $k_{out}$ and the empirical distribution of case study for $k_{in}$. They are combined through a Frank copula. When θ>0, the maximum moves to higher values of θ as *k* increases. Since 0 does not belong to the definition set, there is no minimum. Left side of the figure: in all cases the function is increasing for $\theta \to 0^{+}$ and decreasing for θ→−∞.

**Figure 8:**
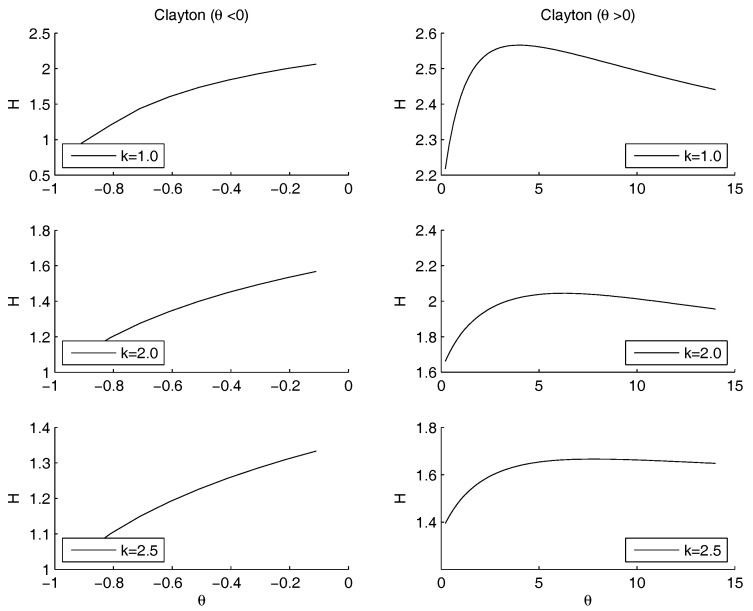
The figure shows three cases for the entropy (y-axis) as a function of θ (x-axis). The marginals are: the power law for $k_{out}$ and from the case study for $k_{in}$. They are combined through a Clayton copula with parameter θ<0. The left figures shows the case θ<0. There is a minimum for θ=−1, for any value of *k*. The right figures show the case θ>0. The maximum moves to the right hand side as *k* increases. For any *k*, the function is decreasing for θ→−∞.

- Non parametric copulas: Figure 5 shows the behavior of the entropy as a function of *k*. The upper Frechet bound and the product copula are quite overlapped: the entropy increases as *k* increases. Practically, in the marginal of $k_{out}$ the entropy is minimal as the mass is pushed to the highest mass concentration of $k_{in}$, that is at the left bound of the domain, although it should not become more sharp than the empirical distribution of $k_{in}$. This is coherent with the Theorem in the Appendix A, as well as with the very well known fact that the entropy is minimal as the dispersion diminishes and the mass is concentrated. The lower Frechet copula has the opposite behavior. There is no minimum and no maximum internal to the range for *k*. All the three show a maximum: for k=0.46 and H=2.12 (Product), k=2.2 and H=0.97 (Lower Frechet), k=1 and H=2.09 (Upper Frechet). The only maximum in the most interesting range of k∈(2,3) is the Upper Frechet one. In the Frechet one there is also another local maximum in k=0.81 and H=0.52 and two local minima in k=0.71 and H=0.50 and in k=0.91 and H=0.48. The other local fluctuations in the Upper Frechet do not lead to other local maxima or minima. All the entropies are decreasing for *k* increasing.
- Figure 6 shows the entropy function when the exponent of the power law for $k_{out}$ is allowed to change. Therefore, the marginal distribution is allowd to change, still remaining a power law. The other marginal is given by the case study for $k_{in}$. The marginal distributions are combined through the Gumbel copula. The minimum that was detected on the raw data for θ=1 disappears, and an asymptotic behavior remains: the entropy is decreasing for θ→∞, i.e., in the case of convergence towards the Frechet upper bound. Therefore, the minimum entropy is obtained either when the copula is the product or when the considered quantities are perfectly positively correlated.
  Once more, we may remark that the entropy decreases as the concentration of the distribution increases, possibly reaching a Dirac’s delta function. Since the marginal on $k_{in}$ is fixed, the minimum is obtained when the mass through the other marginal is concentrated on the highest peak of $k_{in}$, that is at the left border. This effect is obtained by increasing the steepness of the marginal of $k_{out}$. The higher *k*, the more the mass is concentrated on the left border. This effect is emphasized by the application of the copula. Since both marginals are left-skewed, the product gives the minimum, for quite a range of values of *k*. However, the entropy is decreasing as θ→∞, reaching values lower than the minimum, when present. Therefore any concentration limit can be overrun, providing that the slope of the power law is large. We already noted that most systems show a power law with an exponent between 2 and 3. This prevents the rise of concentration.
  The analysis of the maximum is quite different. As *k* increases, the maximum is pushed to the left side of the range of θ, tending to 1 for high values of *k*, i.e., in the case of independence.
- Frank copula. Also for the Frank copula there are different configurations as the parameters of the power law changes. Figure 7 outlines the situation for θ<0 (left hand side) and for θ>0 (right hand side).
  The Frank copula when θ<0 gives a result similar to the left part of the second row of the Figure 4: there is no minimum. Moreover, the value of the entropy is increasing as θ increases. However, for each fixed θ, the values of the entropy decreases as *k* increases. If θ>0 the maximum moves to the right as *k* increases. There is no minimum, since 0 does not belong to the definition set.
- Clayton copula. Figure 8 shows the situation depending on the parameters of the power law. For θ>0, the subplots show that the maximum moves to the right hand side as *k* increases. There is no minimum, since 0 does not belong to the definition set, there is no minimum. For θ<0, there is a minimum for θ=−1, for any value of *k*.

## 6. Conclusions

This paper provides a detailed analysis of the concentration of a market, which is captured by a joint analysis of diversification and integration. Such concepts are strongly linked with the network described by the cross-shareholding matrix and the related entropy measure. In particular, the out-degree value of a company formalizes its diversification while the in-degree value is related to its integration in a network of shareholders. The analysis of such degrees may be relevant for regulatory bodies, that need to fix thresholds and eventually capture early signals for preventing concentration. Literature studies have shown that the most frequently detected probabilities for description of diversification and integration were the power law and the exponential law. The parameters of the distribution regulate their shape. However, it is the coupling between in- and out-degrees which is the most relevant to the concentration evolution.

The dependence between the components of the matrix—the in- and out-degrees—is here captured through appropriately selected copulas. Among them, the most prominent examples of nonparametric copulas—product and Frechet bounds—are also included. The maximum of concentration can be achieved by minimizing the entropy. When one marginal distribution is fixed, the results show that the minimal entropy is achieved when the other marginal distributions gather at the center of mass of the reference marginal distribution. On the opposite, the possibility to reach the maximum disorder of the system strictly is affected by the dependence structure between the in- and out-degree; such an aspect is captured through suitable copulas.

Therefore, the present paper adds new perspectives to some specific aspects of the existing literature. First, portfolio owners are not considered as external to the market, but they are part of the market. This implies the introduction of the concepts of integration and diversification; such an approach creates a bridge between the literature on companies performances and the one on companies interactions, where the embedding of a company in a network is a key factor. Second, we base our analysis on data available both in literature and on the case study for exploring the configurations that lead to max/min entropy when both integration and diversification are considered. Concentration is here intended as the maximal correlation among diversification and integration. It differs from the well known assortativity on networks due to the way of measurement: the assortativity is the correlation among diversification and integration measured from raw data [4]. Differently, concentration is calculated through the entropy and under the hypotheses of different correlation structures, expressed through copulas.

Moreover, the proposed analysis goes in the direction of policy implementation for shaping the market by tending towards a more evident polarization of to a fair distribution of in- and out-degrees. The maximum level of polarization is associated to a monopolistic structure, which represents the desired target when the aim is to totally remove market competition; conversely, the uniform distribution associated to the maximum level of entropy is the scope of a policymaker aiming at fostering the competition between the companies populating the market.

Please note that the analysis could be further enlarged by including other companies variables in the definition of market concentration. In this respect, one can reasonably follow a *n*-variate copula approach with n>2.

## Figures and Tables

**Figure A1:**
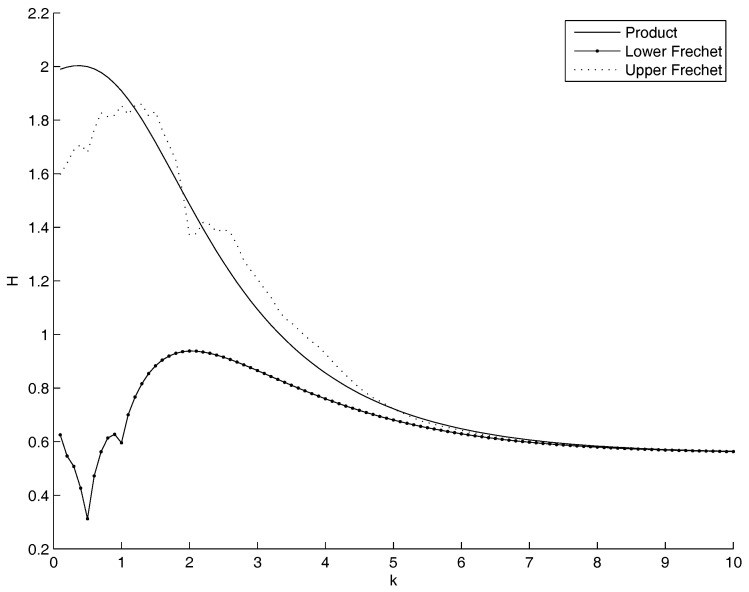
The figure shows three cases for the entropy (y-axis) as a function of θ (x-axis). The marginals are: the power law for $k_{in}$ and from the case study for $k_{out}$. The situation is quite similar to the one in Figure 5, but there are many more local fluctuations in the Upper Frechet copula.

**Figure A2:**
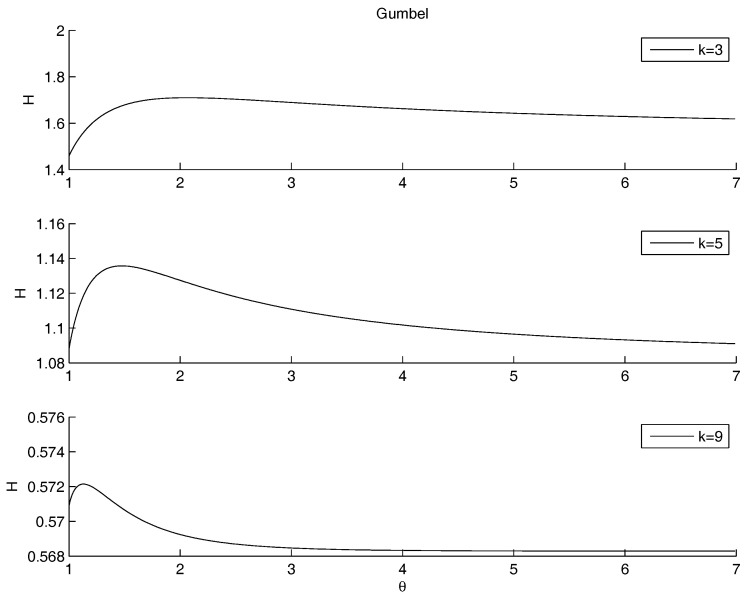
Plot of the dependence of the entropy function *H* on the parameter θ. The same comments as for Figure 6 hold.

**Figure A3:**
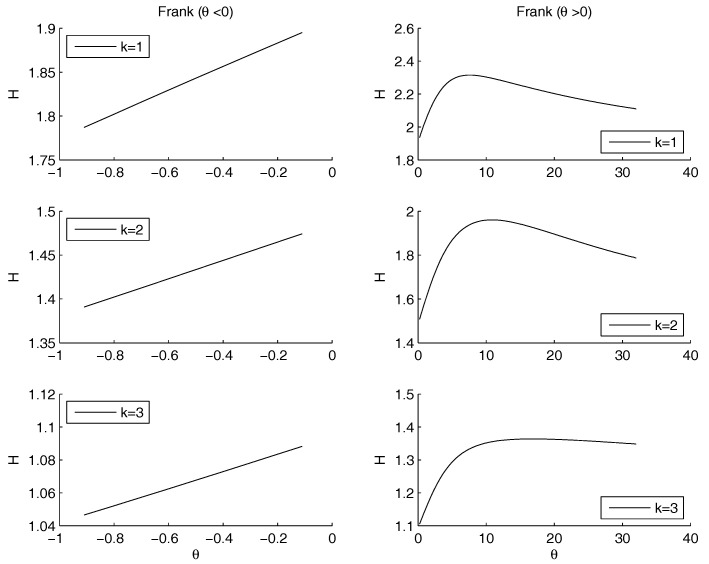
Plot of the dependence of the entropy function *H* on the power law exponent *k*. The same comments as for Figure 7 hold.

**Figure A4:**
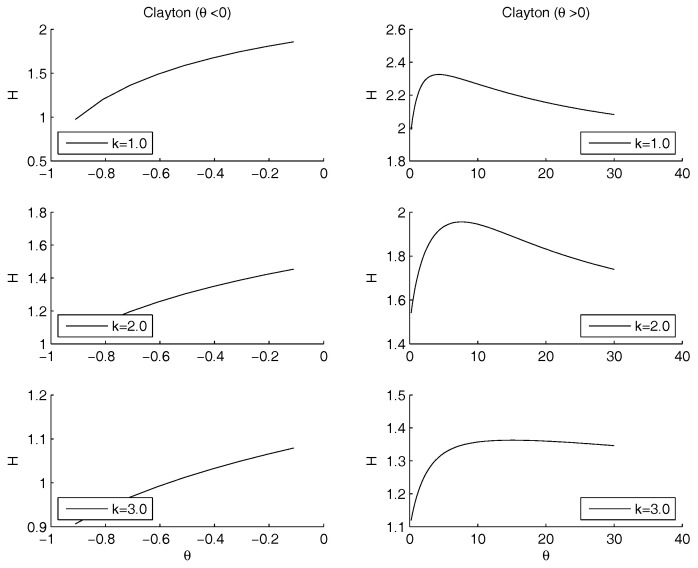
Plot of the dependence of the entropy function *H* on the power law exponent *k*. The same comments as for Figure 8 hold.

**Figure A5:**
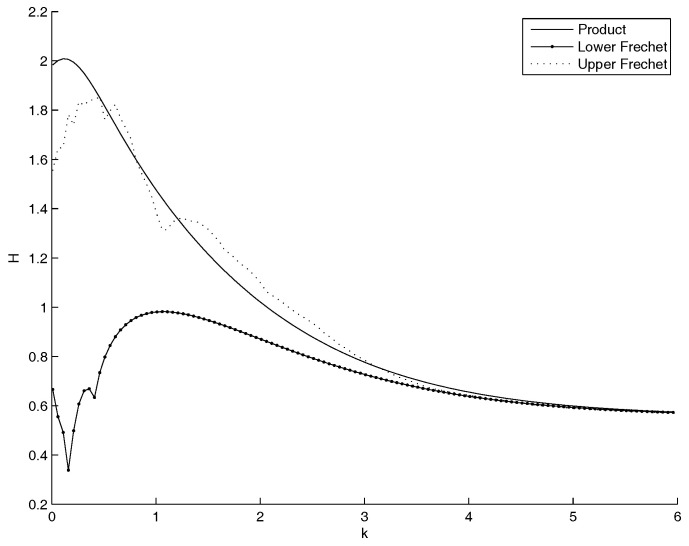
The figure shows three cases for the entropy (y-axis) as a function of θ (x-axis). The marginals are: the power law for $k_{in}$ and from the case study for $k_{out}$. The situation is quite similar to the one in Figure A1, but the local fluctuations in the upper Frechet copula are deeper.

**Figure A6:**
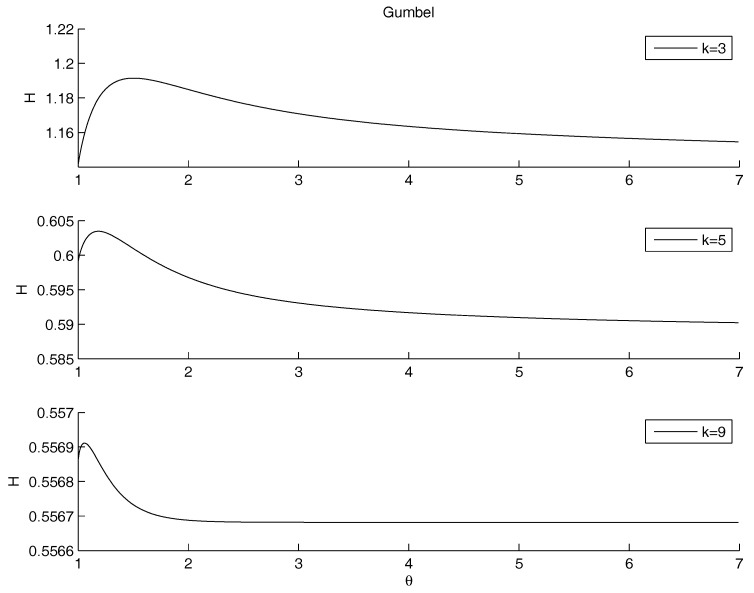
Plot of the dependence of the entropy function *H* on the parameter θ. The same comments as for Figure A2 hold.

**Figure A7:**
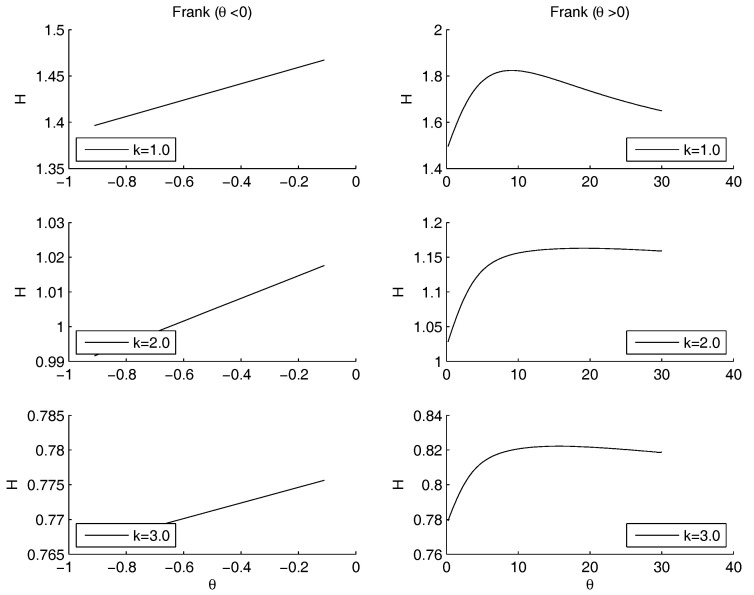
Plot of the dependence of the entropy function *H* on the parameter θ. The same comments as for Figure 7 and Figure A3 hold.

**Figure A8:**
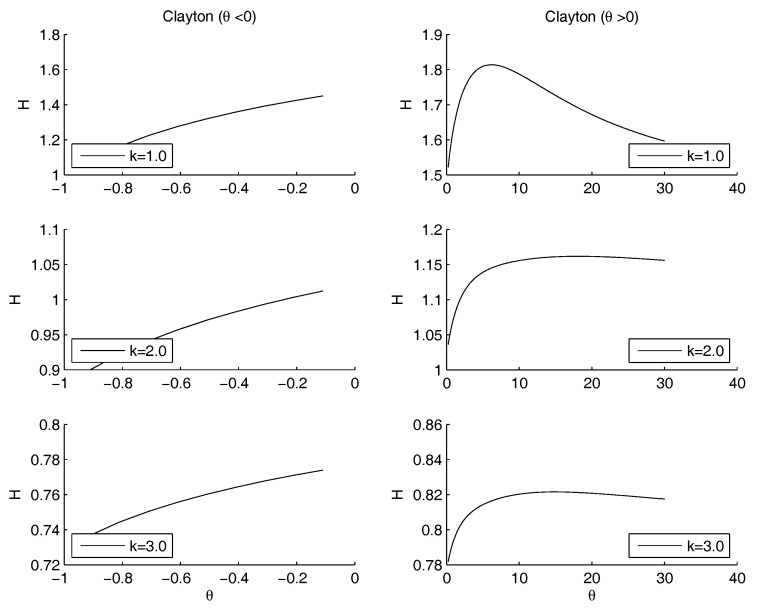
Plot of the dependence of the entropy function *H* on the parameter θ. The same comments as for Figure 7 and Figure A4 hold.

## Appendix A. Maximum of a Product and Minimum of the Shannon Entropy

**Theorem** **A1.** (a) Given two vectors with non negative components $p=(p_{1},p_{2},\cdots ,p_{n})$, $q=(q_{1},q_{2},\cdots ,q_{n})$, then the minimum of the scalar product under the permutation of the components of one of the vectors is achieved for $q^{☆}=\left(q_{1}^{☆},q_{2}^{☆},\cdots ,q_{n}^{☆}\right)$ i.e.: $min_{\pi \in \Pi_{n}}\sum_{k=1}^{n}p_{k}q_{\pi_{k}}=\sum_{k=1}^{n}p_{k}q_{k}^{☆}$, with reverted ranked components, i.e., $p_{i}\geq p_{j}$ and $q_{i}^{☆}\leq q_{j}^{☆}$, for each i<j. (b) Given two vectors with non negative components $p=(p_{1},p_{2},\cdots ,p_{n})$, $q=(q_{1},q_{2},\cdots ,q_{n})$, then the maximum of the scalar product under the permutation of the components of one of the vectors is obtained for $q^{☆}=\left(q_{1}^{☆},q_{2}^{☆},\cdots ,q_{n}^{☆}\right)$ i.e.: $max_{\pi \in \Pi_{n}}\sum_{k=1}^{n}p_{k}q_{\pi_{k}}=\sum_{k=1}^{n}p_{k}q_{k}^{☆}$, with components ranked in the same order, i.e., $p_{i}\leq p_{j}$ and $q_{i}^{☆}\geq q_{j}^{☆}$, for each i<j.

**Proof.** We report only the proof of (a), since the proof of (b) is analogous. (a) It holds $\sum_{k=1}^{n}p_{k}q_{\pi_{k}}=\sum_{k=1,k\neq i,j}^{n}p_{k}q_{\pi_{k}}+p_{i}q_{i}+p_{j}q_{j}\leq \sum_{k=1,k\neq i,j}^{n}p_{k}q_{\pi_{k}}+p_{i}q_{j}+p_{i}q_{j}$. In fact, $p_{i}q_{i}+p_{j}q_{j}\leq p_{i}q_{j}+p_{j}q_{i}$ is equivalent to writing $p_{i}\left(q_{i}-q_{j}\right)-p_{j}\left(q_{i}-q_{j}\right)\leq 0$, that happens when $\left(p_{i}-p_{j}\right)\left(q_{i}-q_{j}\right)\leq 0$, that is verified if, anytime $p_{i}\geq p_{j}$, then $q_{i}\leq q_{j}$. ☐

**Remark** **A1.** Results of Theorem A1 hold under the same hypothesis and for monotonic transformations of p or q. In particular, this is true in case of logarithmic transformation. Now, entropy can be seen as the inner product of two vectors: one containing the probability, and the other its logarithm. Thus, the ranking of the two vectors is always the same, and Theorem A1 guarantees that entropy is maximal when the distributions are as flat as possible, and minimal when the mass is concentrated as most as possible on some units - attaining the true maximum for the Dirac’s Delta function.

## Appendix B. Steps 2-5 of case 3

### Appendix B.1. Step 2: Power Law for k_in_, and Raw Data for k_out_

- Non parametric copulas. The situation is quite similar to Figure 5. The product copula and the Upper Frechet are quite close each to the other. The same comments as for Figure 5 hold. The functions are decreasing as *k* increases. There are local maxima: in k=0.5 H=1.99 (Product), in k=2 H=0.93 (Lower Frechet), in k=1.3 H=1.86 (Upper Frechet). We remark that there are many more small fluctuations, that lead to local minima for the Upper Frechet - although the values of the entropy there is much higher than the value on the tail. In the lower Frechet we remark that the local minima have a different location: for k=1 H=0.59 and or k=0.5 H=0.31. There is also a local maximum in k=0.9 H=0.62

- Gumbel Archimedean copula. Figure A2 shows the case. The same comments as for Figure 6 hold.

- Frank Archimedean copula. Figure A3 shows the case. The same comments as for $k_{in}$ from the empirical data and $k_{out}$ power law hold (Figure 7).

- Clayton Archimedean copula. Figure A4 shows the case. The same comments as for $k_{in}$ from the empirical data and $k_{out}$ power law hold (Figure 8).

### Appendix B.2. Step 3: Exponential Law for k_in_, Raw Data for k_out_

- Non parametric copulas. The situation is quite similar to Figure 5. The product copula and the Upper Frechet are quite close to each other. The same comments as for Figure 5 and Figure A1 hold. The functions are decreasing as *k* increases. Figure A5 shows the results. There are local maxima: in k=0.11 H=2 (Product), in k=1.06 H=0.98 (lower Frechet), in k=0.46 H=1.85 (upper Frechet). We remark that there are many more small fluctuations, that lead to local minima for the upper Frechet—although the values of the entropy there is much higher than the value on the tail. Compared to Figure 9, the local minimum in the upper Frechet at k=1.06, H=1.31 is much deeper, and could be considered a true local minimum. In the lower Frechet case, we remark that the local minima have a different location: for k=0.41 H=0.63 and or k=0.16 H=0.33.
  There is also a local maximum in k=0.31 H=0.66

- Gumbel Archimedean copula. Figure A6 shows the case. The same comments as for Figures 6 and 10 hold.

- Frank Archimedean copula. Figure A7 shows the case. The same comments as for Figure 7 and Figure A3 hold.

- Clayton Archimedean copula. Figure A8 shows the case. The same comments as for Figure 7 and Figure A4 hold.

### Appendix B.3. Steps 4 and 5: either Power Law or Exponential Law for k_in_, and Power Law for k_out_

On the parametric copulas, in view of the numerical results already obtained, of the Theorem A1, and due to Remark A1 in the Appendix, in cases of either power law or exponential law for $k_{in}$, while $k_{out}$ remains described by a power law, we conclude that the entropy diminishes as the parameters for the power law(s) or the exponential go to infinity. There will be local maxima that will go either to the left or to the right border of the range of θ as the power law/exponential parameters increase.
